# Hyperbaric oxygen effectively addresses the pathophysiology of long COVID: clinical review

**DOI:** 10.3389/fmed.2024.1354088

**Published:** 2024-02-15

**Authors:** Alan A. Katz, Sandra Wainwright, Matthew P. Kelly, Pradeep Albert, Rosemary Byrne

**Affiliations:** ^1^Hyperbaric Medical Solutions, New York, NY, United States; ^2^Orlando College of Osteopathic Medicine, Winter Garden, FL, United States; ^3^Greenwich Hospital, Yale New Haven Health System, New Haven, CT, United States; ^4^University of Alabama at Birmingham, Heersink School of Medicine, Birmingham, AL, United States

**Keywords:** long COVID, hyperbaric, hyperbaric oxygen therapy, post-COVID-19 syndrome, HBO

## Abstract

**Background:**

The World Health Organization defines long COVID as “the continuation or development of new symptoms 3 months after the initial SARS-CoV-2 infection, with these symptoms lasting for at least 2 months with no other explanation.” Estimations of approximately 50 million individuals suffer from long COVID, reporting low health-related quality of life. Patients develop ongoing persistent symptoms that continue for more than 12 weeks that are not explained by another alternative diagnosis. To date, no current therapeutics are effective in treating the underlying pathophysiology of long COVID.

**Discussion:**

A comprehensive literature search using PubMed and Google Scholar was conducted and all available articles from November 2021 to January 2024 containing keywords long covid and hyperbaric oxygen were reviewed. These published studies, including case series and randomized trials, demonstrate that utilizing Hyperbaric Oxygen Therapy (HBO) provided significant improvement in patients with long COVID.

**Conclusion:**

A large cohort of patients suffer from long COVID or post-COVID-19 syndrome after recovery from their acute infection with no effective treatment options. HBO is a safe treatment and may provide benefit for this population and should continue to be researched for adjunctive treatment of long COVID.

## Introduction

As the worldwide COVID epidemic continues, a large cohort of patients suffer from long COVID or post-COVID-19 syndrome after recovery from their acute infection (1). Estimations of approximately 50 million individuals (2), or 10–20% of patients initially diagnosed (3), suffer from long COVID reporting low health related quality of life. The World Health Organization defines long COVID as “the continuation or development of new symptoms 3 months after the initial SARS-CoV-2 infection, with these symptoms lasting for at least 2 months with no other explanation” (4). Patients develop ongoing persistent symptoms including dyspnea, cough, fatigue, “brain-fog,” cognitive dysfunction, anxiety, depression, sleep disturbances, palpitations, postural tachycardia syndrome (POTS), and rashes that continue for more than 12 weeks not explained by another alternative diagnosis. Decreased exercise capacity, hypoxia, reduced diffusion capacity, restrictive pulmonary physiology, ground-glass opacities, and fibrotic changes on imaging have been noted after initial COVID infection has resolved (1). Thromboembolic events, hair loss and renal impairment have all been noted in follow up. Symptoms can be severe and hinder productivity, most often in economically active adults (1, 5).

Post-COVID syndrome is well described worldwide with symptoms affecting quality of life and productivity. To date, no current therapeutics are effective in treating the underlying pathophysiology of long COVID. Recent studies, including case series and randomized trials, demonstrate that Hyperbaric Oxygen Therapy (HBO) treated patients had significant improvement in global cognitive function, fatigue, attention, executive function, energy, sleep, psychiatric symptoms, cardiopulmonary function, endurance and pain. HBO is beneficial and safe to treat patients with long COVID.

## Discussion

### Presentation and pathophysiology of long COVID

An observational cohort study from 38 hospitals in Michigan evaluated the outcomes of 1,250 patients through record review and telephone surveys. 488 patients completed the telephone survey with 32.6% of patients reporting persistent symptoms, including dyspnea while walking up the stairs (22.9%), cough (15.4%) and persistent loss of taste and/or smell (13.1%) (6). The CDC, in a multivariate regression model, studied adults and found that the risk of developing long COVID was higher in those in the age range of 40–54, female, with co-morbidities, and black people. The results of a sample size of 366 people are consistent with clinical observations. The economic impact of removing people who otherwise would be at the peak of their productive years is profound (7).

The precise pathophysiology of long COVID is unknown and may vary between individuals. Symptoms are thought to be related to possible auto-immune disease due to dysregulated T-cell activation, chronic inflammation, chronic oxidative stress, mitochondrial dysfunction, endothelial dysfunction, thrombotic disease, tissue hypoxia, and direct brain invasion by the virus (8, 9). In a recent prospective study, a cohort of 31 patients who reported the presence of one of the following symptoms: dyspnea, fatigue, chest pain, were matched with 31 individuals who had prior COVID infection but no evidence of long COVID^10^. Those with long COVID symptoms showed increased frequency of activated CD14 + CD16+ monocytes and plasmacytoid dendritic cells, compared with control individuals (10). The individuals studied demonstrated persistent elevation in the levels of type I (IFNβ) and type III (IFNλ1) interferon 8 months post-infection. The combination of IFNβ, pentraxin 3, IFNγ, IFNλ2/3 and IL-6 was associated with long COVID symptoms, with an accuracy ranging from 78.5 to 81.6% (10). The levels observed have been associated with acute, severe disease, suggesting that the long COVID symptoms are a result of delayed or defective resolution of inflammation (10).

T-cell dysfunction may promote long COVID pathophysiology. Consistently, autopsy examinations of deceased COVID-19 patients demonstrated that infiltrates in the lungs and other organs were enriched with CD8+ T cells (11). Thyroid dysfunction has been detected in 15–20% of patients with COVID-19, suggesting that thyroid effect on T cell-mediated autoimmunity may play a role in the autoimmunity pathophysiology of long COVID (12).

B-cells may also be involved in long COVID autoimmunity. In severe cases of COVID-19, it has been shown that COVID-19 infection causes lymphopenia (i.e., B-cell and T-cell lymphocytes deficiency) that causes hyperinflammation (12). Subsequently, as B-cell and T-cell lymphocytes are renewed, elevated inflammation may develop, leading to symptoms of long COVID (12).

Elevated IL-6 levels have been observed in severe and moderate cases of COVID-19 infection causing inflammation and oxidative stress resulting from excessive reactive oxygen species (ROS) production and depleted antioxidant systems (13). Because inflammation and oxidative stress mutually reinforce one another, the elevation of IL-6 and ROS leads to a state of hyperinflammation post COVID infection (14).

### Potential treatment options

Studied treatments for long COVID include anti-inflammatory agents, specific diets, cognitive behavioral therapy, rehabilitation, and hyperbaric oxygen therapy (15, 16). No universally effective treatments for long COVID have been identified. However, treatments aimed at symptom categories have shown efficacy in certain groups, such as pharmacological options targeting symptoms such as β-blockers for POTS, low-dose naltrexone for neuroinflammation and intravenous immunoglobulin for immune dysfunction. H_1_ and H_2_ antihistamines may relieve symptoms involving mast cell activation and anticoagulant regimens can counteract abnormal clotting (17). Many non-pharmacological options have been utilized including cognitive pacing for ME/CFS symptoms, increasing salt intake and compression stockings for POTS, and probiotics and elimination diets for gastrointestinal symptoms (17). Some supplements have shown promise in treating long COVID including coenzyme Q_10_ and d-ribose (17).

### Mechanism of action and rationale for the use of HBO in long COVID

The mechanism of action of HBO involves both increased pressure and elevated partial pressure of O_2_. The former causes a reduction of bubble size related to Boyle’s Law however much of the clinical efficacy of HBO is derived from the high O_2_ partial pressures and hyperoxia that increase the production of reactive O_2_ species (ROS) and of reactive nitrogen species (RNS). HBO promotes the synthesis of growth factors and mitigates post-ischemic and post-inflammatory responses (18).

HBO also effects the expression of immune-modulatory cytokines by decreasing proinflammatory cytokines such as IL-1, IL-6, and TNF-α and elevating the anti-inflammatory cytokine IL-10 (19). Many HBO protocols call for the intermittent fluctuation of O_2_ levels (from 100 to 20.9% for brief periods). These fluctuations serve to induce the Hyperoxic-Hypoxic Paradox which increases oxidative stress scavenger transcription factors and subsequently increases the production of antioxidant enzymes (20). HBO elevates ROS productions, especially via mitochondrial function but also elevates antioxidant levels and activity, thereby reducing overall ROS level. Conversion of oxygen to ROS is a function of metabolic rate and the mitochondria serve as the main source of oxidative stress. HBO causes an increase in ATP production levels, decreased mitochondria-mediated apoptosis signaling, and reduced mitochondrial membrane potential. It has become clearer that mitochondrial dysfunction drives many disease processes, so the effects of HBO on oxidative phosphorylation and ROS likely contribute to its therapeutic benefits (20).

Long COVID pathophysiology is characterized by dysregulated T-cell activation, chronic inflammation, chronic oxidative stress, mitochondrial dysfunction, endothelial dysfunction, thrombotic disease, and tissue hypoxia (8, 9). The beneficial effects of HBO on mitochondrial function likely contribute to the mechanism of action when treating many of the symptoms of long COVID. Another possible mechanism of action of HBO in the treatment of long COVID is reduced production of proinflammatory cytokines (21). HBO increases the mobilization of stem cells (21). It is through this mechanism that HBO can inhibit the abnormal activation of T lymphocytes and macrophages and decreases the secretion of proinflammatory cytokines. HBO provides benefits to sufferers of long COVID through the enhanced mitochondrial function, reduction in inflammation, mobilization of stem cells, improvement in thrombotic disease and the relief of hypoxia (21) (see Table 1).

**Table 1:** HBO for long COVID utility.

Physiologic mechanism	Long COVID	Hyperbaric oxygen
Inflammation	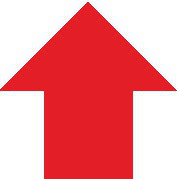	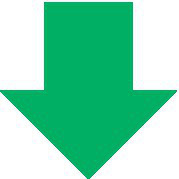
Prothrombotic/ischemic	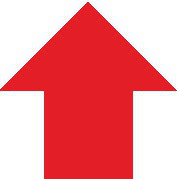	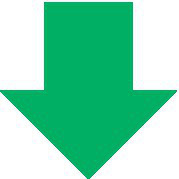
Inflammatory cytokines IL-1, IL-6, TNF-α	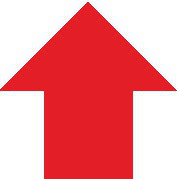	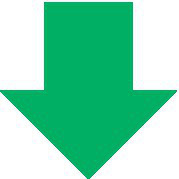
Anti-inflammatory cytokine IL-10	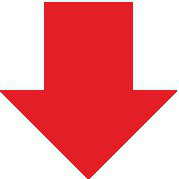	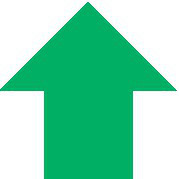
ATP production	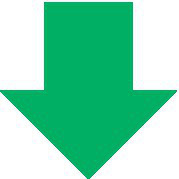	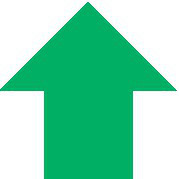
Mitochondrial apoptosis signaling	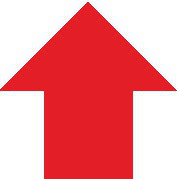	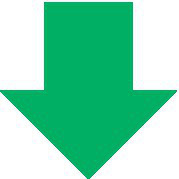
Dysregulation of T-cells	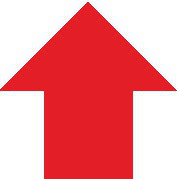	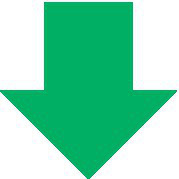
Endothelial dysfunction	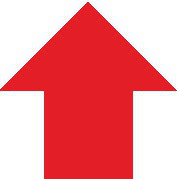	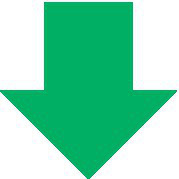
Tissue hypoxia	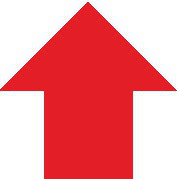	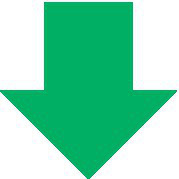

### Review of literature regarding use of HBO in long COVID

HBO has been studied for patients with long COVID syndrome. A comprehensive literature search using PubMed and Google Scholar was conducted and all available articles from November 2021 to January 2024 containing keywords “long covid” and “hyperbaric oxygen” were reviewed.

In a trial of six patients with long COVID symptoms treated with HBO, 6/6 patients saw improvement in symptoms, 5/6 of whom returned to pre-infection levels of illness (22). All the patients studied had developed dyspnea symptoms in the slight to moderate range of the modified Borg scale (average dyspnea score: 3.81). After completing 15 to 29 HBO treatments, dyspnea scores were significantly reduced (average dyspnea score post-HBO2: 0.17) in all patients (22). A case series of 10 patients treated with HBO yielded statistically significant improvements in fatigue, global cognition, executive function, attention, information processing speed, and verbal function (23). No adverse effects of HBO on these patients were noted.

Zilberman-Itskovich et al. in a randomized, sham-controlled, double-blind trial recently reported similar results (24). Seventy-three patients were randomized to receive HBO treatments vs. sham treatment. These patients were treated with HBO for 40 sessions. HBO treated patients had significant improvement in global cognitive function, attention, executive function, energy, sleep, psychiatric symptoms, and pain. Improvements in brain MRI perfusion and microstructural changes were noted, highlighting HBO’s beneficial effect on inducing neuroplasticity (25).

An on-going Swedish study looking at HBO for long COVID (HOT-LoCO) recently published an interim safety report from their ongoing trial, reporting mostly mild adverse events, indicating that HBO can be safely utilized in long COVID patients; outcome measures have not yet been reported (26).

A recent randomized, sham-controlled, double-blind trial addressed the effects of long COVID on cardiac dysfunction. Sixty patients who demonstrated ongoing left ventricular dysfunction symptoms for at least three months after COVID infection were randomized to receive 40 HBO or sham sessions. Echocardiography was performed at baseline and 1–3 weeks after the last HBO session. Twenty-nine (48.3%) patients had reduced global longitudinal strain (GLS) at baseline. Compared to the sham group, GLS significantly increased following treatment with HBO, illustrating that HBO enhances left ventricular systolic function recovery in patients suffering from long COVID-19 induced subclinical left ventricular dysfunction (27).

A recent trial evaluated oxy-inflammation biomarkers in long COVID-19 subjects treated with HBO. The study examined five subjects who received 100% O2 at 2.4 ATA for 90 min. Three of the patients received 15 sessions, one received 30 sessions and one received 50 sessions, with daily sessions, 5 times per week. Reactive oxygen species (ROS), antioxidant capacity, cytokines, lipids peroxidation, DNA damage, and renal status were assessed pre-treatment and after completion of HBO. The data showed reduction of ROS production, lipid peroxidation and DNA damage. There was a reduction of nitric oxide metabolites and inflammatory biomarkers (28). The results demonstrate that HBO may effectively mitigate the COVID-19-induced inflammation.

A prospective trial published in 2022 treated 31 patients with 15 sessions at HBO, reporting significant and sustained improvement in quality of life, endurance and strength, spirometry parameters, and working memory and attention (29).

A published case report of a 55-year-old male who received HBOT with pre and post perfusion MRI also demonstrated significant improvements in brain perfusion, white matter brain microstructure, and cognitive and cardiopulmonary function (30) (see Table 2).

**Table 2:** Summary of reported trials.

Author/Year	*N*	Trial design	Biomarkers/testing	Scales	Treatment profile	Results	Outcome
Kjellberg et al. (26)	20	Randomized double blind placebo	n/a	RAND-36 6MWT	2.4 ATA 90 min, two 5-min air breaks × 40 sessions	Trial design, no serious adverse events, favorable safety profile	SAFE
Robbins et al. (23)	10	Case series	NeuroTrax	Chalder fatigue scale	2.4 ATA 105 min, 3, 5-min air breaks × 10 sessions over 12 days	Improvement in fatigue and cognitive measures	+
Leitman et al. (27)	79	Randomized controlled	Echocardiogram	n/a	2.0 ATA 90 min, three 5-min air breaks × 40 sessions	48% of long COVID patients demonstrated pre-HBOT systolic dysfunction which was significantly improved with HBOT	+
Lindenmann et al. (31)	59	Prospective	n/a	SF-36 VAS	2.2 ATA 75 min × 10 sessions (no air breaks mentioned)	In as little as 10 HBOTs—statistical improvement in 80% of metrics, safe and feasible tool for LCS	+
Zant et al. (22)	6	Clinical case report	ImPACT	Modified Borg dyspnea scale	2.0 ATA 90 min × 15–29 sessions (no air breaks mentioned)	All patients saw improvement in symptoms scores	+
Kitala et al. (29)	31	Prospective	Pulse oximetry, spirometry	ROM, EQ-5D-5L psychotechnical test	2.5 ATA 75 min × 15 sessions (no mention of air breaks)	HBOT resulted in significant and lasting improvement in QOL, endurance, strength, spirometry, memory and attention	+
Zilberman-Itskovich et al. (24)	73	Prospective randomized sham-controlled	Voxel based neuroimaging	SF-36, PSQI, BSI-18	2.0 ATA 90 min, three 5-min air breaks × 40 sessions	HBOT improves cerebral blood flow and brain microstructural changes in those areas that are associated with executive function, cognitive and psychiatric symptoms	+
Mrakic-Sposta et al. (28)	5	Case series	ROS, TAC, (IL-6, IL1β and TNF-α) lipid peroxidation, DNA damage, No metabolites, neopterin, creatinine, uric acid, spirometry	Fatigue scale	2.4 ATA 90 min (no mention of air break) *N* = 2, 15 sessions *N* = 2, 30 sessions *N* = 1, 50 sessions	Statistically significant effect of HBOT on biomarkers in all subjects. HBOT is a potential *treatment* for long COVID patients	+
Bhaiyat et al. (30)	1	Case report	Perf MRI, Exercise, spirometry	n/a	2.0 ATA 90 min, three 5-min air breaks × 60 sessions	Improved cognition and cardiopulmonary function	+
Kjellberg et al. (32)	n/a	Safety analysis of HOT-LoCO	n/a	RAND-36 6MWT	2.4 ATA 90 min, two 5-min air breaks × 10 sessions	Trial design, safety assessment and rationale for HBOT and future study	SAFE

## Conclusion

Long COVID-related effects can be debilitating and often affect people who are economically productive. Eight published studies show that HBO has significant effects in improving the lives of patients diagnosed with long COVID. There are no other treatment options currently available that improve symptoms. HBO directly addresses the pathophysiology of long COVID including chronic inflammation, small vessel injury, disrupted neural pathways and mitochondrial dysfunction. There is increasing evidence which supports the use of HBO in treating patients suffering from the effects of long COVID. HBO has been documented as safe to use in patients suffering from long COVID. HBO may provide benefit to those suffering from long COVID symptoms and should continue to be researched for adjunctive treatment of long COVID.

## Author contributions

AK: Conceptualization, Writing – original draft, Writing – review & editing. SW: Writing – original draft, Writing – review & editing. MK: Writing – original draft. PA: Writing – original draft. RB: Writing – original draft, Writing – review & editing.
